# Side-Specific Concordance Between Nasal Endoscopy and Computed Tomography Severity in Pediatric Chronic Rhinosinusitis

**DOI:** 10.3390/diagnostics16152326

**Published:** 2026-07-24

**Authors:** Anna Kasprzyk, Ewa Franczak-Józefkiewicz, Grażyna Mielnik-Niedzielska, Artur Niedzielski

**Affiliations:** 1 Individual Specialist Medical Practice, 03-736 Warsaw, Poland; 2 Department of Pediatric Otolaryngology, Phoniatrics and Audiology, Medical University of Lublin, 20-093 Lublin, Poland; 3 Department of Medical Education, Centre of Postgraduate Medical Education, 01-813 Warsaw, Poland

**Keywords:** chronic rhinosinusitis, child, nasal endoscopy, computed tomography, Lund–Kennedy score, Lund–Mackay score

## Abstract

**Background/Objectives**: The European Position Paper on Rhinosinusitis (EPOS) diagnoses chronic rhinosinusitis (CRS) from symptoms with nasal endoscopy and/or computed tomography (CT), but how closely a graded endoscopic score reflects CT severity in children—and whether it does so side-specifically—is poorly defined. We quantified this concordance to confirm when CT might be deferred. **Methods**: Retrospective analysis of 73 children (33 girls, 40 boys; aged 5–17 years) treated surgically for CRS after failed medical therapy. CT was scored with the Lund–Mackay scale (LMS); a modified score (MLMS) accounting for sinus underdevelopment was also computed. Nasal endoscopy, performed by the same examiner the day before surgery, was scored separately for each side with the Lund–Kennedy system (discharge, edema and polyps, each 0–2; side score 0–6; total 0–12). Concordance was assessed with Spearman correlations (cluster bootstrap for side-level data), Cohen’s kappa and sensitivity/specificity and Bland–Altman analysis after normalization to percentage of maximum. **Results**: Endoscopy correlated strongly with CT severity at the patient level (endoscopy total vs. LMS ρ = 0.76, *p* < 0.0001) and side-specifically (146 sides; ρ = 0.63, *p* < 0.0001); mucosal edema was the strongest individual sign. Bland–Altman analysis showed a systematic bias (endoscopy −10.6% relative to CT) and wide 95% limits of agreement (−32.6% to +11.4%), indicating that endoscopy ranks disease severity well but is not quantitatively interchangeable with CT at the individual level. Dichotomized side-specific agreement was more modest and improved as the CT threshold for a diseased side was raised (κ 0.21–0.50). Symptom burden correlated moderately with CT severity (ρ = 0.41). Children with polyps had higher LMS than those without (*p* = 0.007). Adenoid size, asthma and allergy were unrelated to CT severity. Only the agger nasi cell correlated with ipsilateral frontal disease (adjusted *p* = 0.017). **Conclusions**: A graded nasal endoscopic score is a strong ordinal correlate of CT severity in pediatric CRS, but Bland–Altman analysis shows it cannot quantitatively substitute for CT at the individual level. Endoscopy is therefore best used as a non-ionizing surrogate to grade severity, monitor disease and select which children need CT, while CT remains necessary for surgical planning. Because the cohort comprised children with advanced CRS, generalizability to milder disease requires confirmation.

## 1. Introduction

Chronic rhinosinusitis (CRS) is a persistent inflammatory disorder of the mucosa lining the nasal cavity and paranasal sinuses. Because the mucosa forms an anatomical and functional continuum, the term rhinosinusitis is preferred to sinusitis [1,2]. The disease is classified by symptom duration: acute rhinosinusitis resolves within 12 weeks, whereas in CRS the inflammatory process persists beyond 12 weeks without a complete symptom-free interval [3].

According to the European Position Paper on Rhinosinusitis and Nasal Polyps (EPOS 2020), the diagnosis of CRS rests on two or more compatible symptoms together with objective evidence of inflammation obtained from nasal endoscopy and/or computed tomography (CT) [3]. These three elements—symptoms, endoscopy and CT—are the pillars of diagnosis, and each has limitations in children. Symptoms are non-specific and overlap with adenoid hypertrophy, allergy and recurrent viral infection [3,4,5]. Nasal endoscopy is non-invasive and visualizes mucosal swelling, discharge, polyps, adenoid size, turbinate hypertrophy and septal deviation, but only the accessible nasal surfaces are operator-dependent [3]. CT with multiplanar reconstruction remains the reference standard for imaging [6,7], yet it delivers ionizing radiation to a radiosensitive population, and incidental mucosal abnormalities are extremely common: in children without sinus disease, Hill et al. found that only 19.3% had entirely normal sinus imaging [8].

Despite the central role of these assessments, how closely nasal endoscopy reflects CT severity in children—and whether it does so side by side—has rarely been quantified. A clinically actionable question is whether a graded endoscopic score is sufficiently informative to defer or select CT in pediatric CRS, given the radiation cost in children. The radiological grading of severity is itself difficult: the Lund–Mackay scale (LMS) is the most widely used CT scoring system [9], but it was designed for adults and does not account for incomplete sinus pneumatization in childhood [10,11,12,13], which has prompted proposals for modified, developmentally adjusted scores [10,11,14].

Recent updates to pediatric CRS management [15] have re-emphasized the value of structured endoscopic assessment, and graded endoscopic scoring has been validated repeatedly in adults; pediatric data on the direct concordance of a graded endoscopic score with CT severity, however, remain sparse, particularly at the level of individual sides.

The present study had two aims. The primary aim was to quantify the side-specific and patient-level concordance between a graded nasal endoscopic score (Lund–Kennedy system) and CT severity in children with CRS, thereby informing the clinical decision of when CT might be deferred in favor of endoscopic assessment. The secondary aim was to examine symptom burden, adenoid size, mucosal histopathology, atopy-related comorbidities and anatomical variants in relation to disease severity, and to compare a modified Lund–Mackay score with the conventional scale.

## 2. Materials and Methods

### 2.1. Study Design and Patients

**Figure 1:**
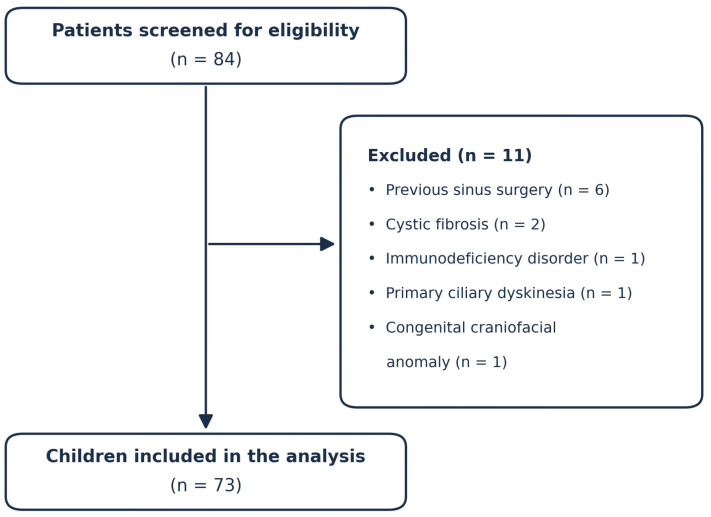
Flow of patients through the study. Of 84 children screened for eligibility, 11 were excluded for the reasons shown, leaving 73 children in the analyzed cohort.

This retrospective study analyzed children with CRS who had failed at least three months of conservative therapy at the Department of Pediatric Otolaryngology, Phoniatrics and Audiology, University Children’s Hospital in Lublin, Poland, between January 2012 and December 2017. Eighty-four consecutive patients aged 5–17 years were screened. Conservative therapy comprised, in varying combinations, intranasal corticosteroids, antihistamines, mucolytics, antibiotics and saline irrigation. The exclusion criteria were diagnosed immunodeficiency, congenital craniofacial anomaly, cystic fibrosis, primary ciliary dyskinesia and previous sinus surgery. Eleven patients were excluded—six with previous sinus surgery, two with cystic fibrosis, and one each with an immunodeficiency disorder, primary ciliary dyskinesia and a congenital craniofacial anomaly—leaving 73 children for analysis (Figure 1).

### 2.2. Computed Tomography

**Figure 2:**
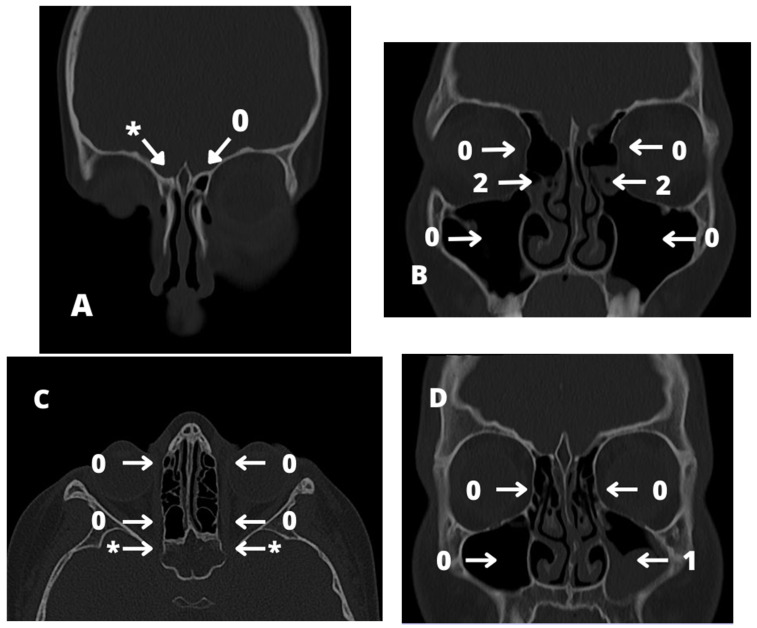
Examples of computed tomography (CT) findings of the paranasal sinuses and assessment of inflammatory disease severity using the Lund–Mackay scoring system. (**A**)—aplasia of the right frontal sinus (marked with *), (**B**)—bilateral obstruction of the osteomeatal complexes, (**C**)—aplasia of both sphenoid sinuses (marked with *), (**D**)—moderate inflammatory changes within the left maxillary sinus.

Sinus CT was performed upon admission or earlier in an outpatient setting (Siemens Somatom Definition AS, Siemens Healthineers, Bavaria, Germany) and reviewed in the coronal, sagittal and axial planes using RadiAnt DICOM Viewer 4.6.5 software. Development of each sinus (frontal, anterior and posterior ethmoid, maxillary and sphenoid, each side) was recorded first. Inflammatory severity was then scored with the Lund–Mackay scale (LMS): each sinus was rated 0 (no opacification), 1 (partial opacification) or 2 (complete opacification), and the ostiomeatal complex was rated 0 (patent) or 2 (obstructed), giving a maximum of 24 points. A per-side CT sub-score (range 0–10) was derived as the sum of the five sinus sub-scores on that side, for comparison with the side-specific endoscopic findings. Representative CT slices illustrating the range of findings are shown in Figure 2.

The same scans were re-scored with a modified Lund–Mackay scale (MLMS) in which underdeveloped sinuses were excluded from scoring and the result rescaled as MLMS = (LMS × 24)/LMSmax, where LMSmax is the maximum attainable score for that patient (24 reduced by 2 for each underdeveloped structure). CT scans were additionally assessed for anatomical variants of the lateral nasal wall—agger nasi cell, concha bullosa (pneumatised middle turbinate), infraorbital (Haller) cell, sphenoethmoidal (Onodi) cell and nasal septal deviation—using current rhinological nomenclature [16]. Septal deviation was considered significant when the septum contacted the middle turbinate or lateral nasal wall or narrowed the middle meatus [17].

### 2.3. Nasal Endoscopy and Adenoid Assessment

**Figure 3:**
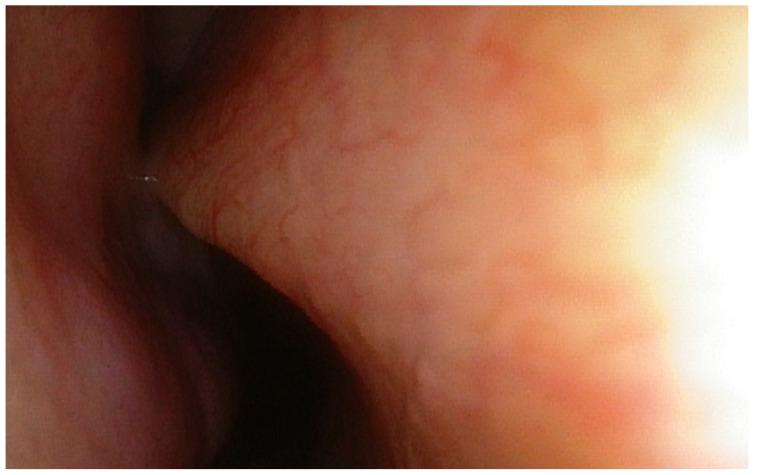
Nasal septal deviation with a right-sided bony septal spur abutting the lateral nasal wall.

**Figure 4:**
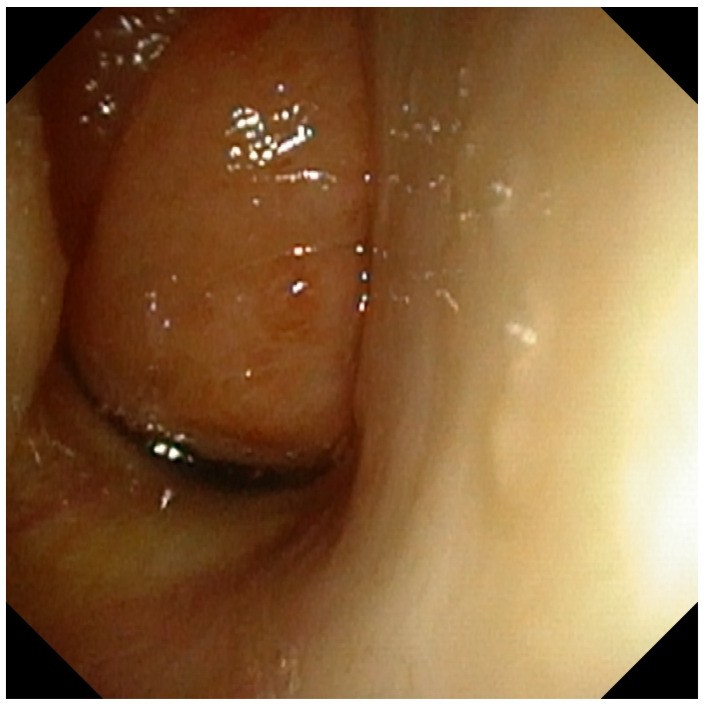
Marked adenoid hypertrophy resulting in near-complete (approximately 100%) obstruction of the choanae.

**Figure 5:**
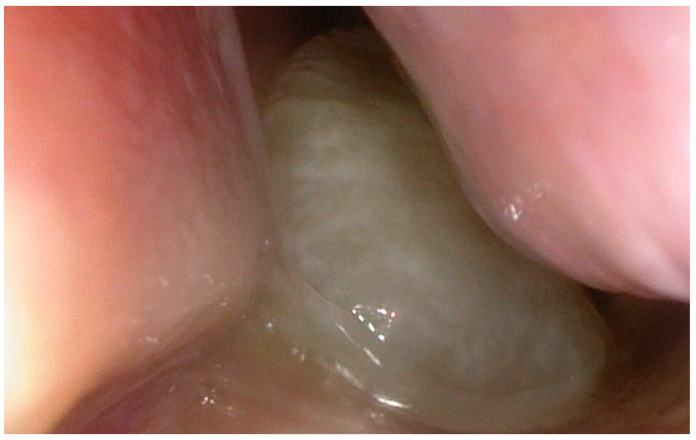
Polyp in the anterior ethmoid air cells.

**Figure 6:**
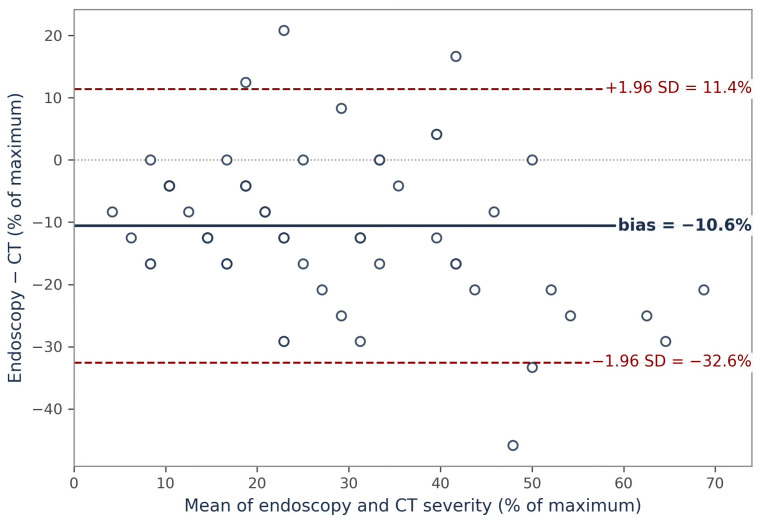
Bland–Altman plot comparing the total Lund–Kennedy endoscopy score with the Lund–Mackay CT score after normalization of each to a percentage of its own maximum (endoscopy 0–12; LMS 0–24). The solid line shows the mean difference (endoscopy − CT = −10.6 percentage points, *p* < 0.0001); the dashed lines show the 95% limits of agreement (−32.6% to +11.4%). Endoscopy systematically underestimates disease severity relative to CT, with wide limits of agreement, indicating that the two scales are not quantitatively interchangeable at the individual level despite a strong rank correlation.

Flexible nasal endoscopy was performed with a fiber-optic nasendoscope (Pentax Medical, Tokyo, Japan, FNL-7RP3, 2.2 mm diameter) on the day of hospital admission, one day before surgery, by the same examiner who scored the CT studies. Topical anesthesia was applied as a lignocaine gel coating the endoscope rather than as a mucosal spray, thereby limiting any direct effect on the appearance of the nasal mucosa. Each side of the nasal cavity was examined and scored separately using the Lund–Kennedy endoscopic system: mucopurulent discharge, mucosal edema and nasal polyps were each graded 0, 1 or 2, giving a side score of 0–6 and a total endoscopy score of 0–12 [18]. Adenoid size was recorded as the percentage of choanal obstruction observed endoscopically. Representative endoscopic images are shown in Figure 3, Figure 4, Figure 5 and Figure 6.

CT scans were performed and clinically reviewed before the endoscopy was undertaken, and the same examiner who interpreted the imaging carried out and scored the endoscopy; no formal blinding was applied. This is acknowledged as a potential source of observer bias and is addressed in Section 4.

### 2.4. Symptoms, Comorbidities and Histopathology

Four cardinal symptoms—nasal discharge, nasal obstruction, headache or facial pressure, and cough—were extracted retrospectively from clinical records and recorded as present or absent; a symptom count (0–4) was derived. A validated pediatric CRS patient-reported outcome instrument was not in routine use at the center during the study period, and the retrospective design precluded its prospective collection. Documented comorbidities—asthma, allergy and passive smoke exposure—were also recorded as present or absent. Mucosal specimens obtained at surgery were classified by the predominant inflammatory infiltrate as eosinophilic, neutrophilic or nonspecific.

### 2.5. Statistical Analysis

Quantitative data are summarized as mean ± standard deviation or median with range. Associations between ordinal or non-normally distributed variables were assessed with the Spearman rank correlation coefficient (ρ); 95% confidence intervals were obtained by bias-corrected and accelerated bootstrap resampling (4000 iterations), with cluster bootstrapping (resampling patients) for side-level data. Agreement between the two continuous scores was quantified with a Bland–Altman analysis after normalizing each score to a percentage of its own maximum (endoscopy 0–12; LMS 0–24); the mean bias and 95% limits of agreement (bias ± 1.96 SD) were computed. Agreement between dichotomized CT and endoscopic classifications was quantified with the observed proportion of agreement and Cohen’s kappa, and the sensitivity and specificity of endoscopy were calculated using CT as the reference. Group comparisons used the Mann–Whitney U test or the Kruskal–Wallis test. The correlations of the endoscopy score with the LMS and MLMS were compared using Williams’ test for dependent correlations. For the eight variant–sinus correlations, *p*-values were adjusted for multiple comparisons by the Benjamini–Hochberg procedure. A two-sided *p* < 0.05 was considered significant.

Analyses were performed using R (R Foundation for Statistical Computing, Vienna, Austria).

### 2.6. Ethics

The study was conducted in accordance with the Declaration of Helsinki. Because of its retrospective design, it was reviewed and approved by the Bioethics Committee of Lazarski University, Warsaw, Poland (approval no. 07/KB/2024, dated 3 March 2024).

## 3. Results

### 3.1. Cohort and CT Findings

Seventy-three children (33 girls, 40 boys) aged 5–17 years (mean 13.2 ± 2.8 years, median 14 years) met the eligibility criteria. All patients had developed maxillary and ethmoid sinuses. The frontal sinus was absent bilaterally in 10 children (13.7%) and unilaterally in a further 6, and the sphenoid sinus was absent bilaterally in 5 children (6.8%).

The maxillary sinus was the most frequently affected, followed by the anterior ethmoid, posterior ethmoid, sphenoid and frontal sinuses (Table 1). The mean LMS was 7.5 ± 4.3 (range 2–19), with 54 children (74.0%) reaching the LMS ≥ 5 threshold, regarded as characteristic of CRS. The mean MLMS was 7.9 ± 4.5 (range 2.0–21.6).

### 3.2. Endoscopic, Symptom and Comorbidity Findings

On nasal endoscopy, mucosal edema was the most common finding, present in 65 children (89.0%) and on 96 of the 146 nasal sides, followed by mucopurulent discharge (40 children, 54.8%) and nasal polyps (7 children, 9.6%). The total Lund–Kennedy endoscopy score (range 0–12) averaged 2.5 ± 1.7 (median 2, observed range 0–7); 69 children (94.5%) had at least one endoscopic abnormality. Adenoid size averaged 26.3 ± 19.8% choanal obstruction (median 20%), exceeding 50% in 12 children (16.4%). The most frequent symptom was nasal discharge (87.7%), followed by nasal obstruction (64.4%), headache (60.3%) and cough (38.4%); the median symptom count was 2 (range 1–4). Documented comorbidities included asthma (6 children, 8.2%), allergy (13, 17.8%) and passive smoke exposure (22, 30.1%). Descriptive findings are summarized in Table 2.

### 3.3. Side-Specific Concordance Between Endoscopy and CT

Across the 146 nasal sides, the per-side Lund–Kennedy endoscopy score correlated strongly with the ipsilateral CT sub-score (Spearman ρ = 0.63, cluster-bootstrap 95% CI 0.50–0.74, *p* < 0.0001). All three endoscopic signs contributed: mucosal edema correlated most closely with ipsilateral CT severity (ρ = 0.53), followed by mucopurulent discharge (ρ = 0.42) and polyps (ρ = 0.35; all *p* < 0.0001). The graded scores varied meaningfully across patients—the LMS spanned 2–19 (SD 4.3) and the Lund–Kennedy total 0–7 (SD 1.7)—so the observed correlation reflects the gradation of disease severity rather than mere co-prevalence. When each side was dichotomized, agreement between endoscopic positivity and CT was more modest and depended on how a diseased side was defined: as the CT threshold was raised, the observed agreement and Cohen’s kappa rose from 71.9% and 0.21 (any opacification) to 77.4% and 0.50 (sub-score ≥ 3) (Table 3). Dichotomizing the scores therefore discards the gradation that carries the diagnostic information.

### 3.4. Patient-Level Concordance and Symptoms

At the patient level, the total endoscopy score correlated strongly with CT severity (vs. LMS ρ = 0.76, 95% CI 0.63–0.86; vs. MLMS ρ = 0.76, 95% CI 0.64–0.85; both *p* < 0.0001). Symptom burden correlated only moderately with CT severity (symptom count vs. LMS ρ = 0.41, 95% CI 0.20–0.59, *p* = 0.0003) and with the endoscopy score (ρ = 0.43, 95% CI 0.21–0.62, *p* = 0.0002); mean LMS rose with symptom count, from 6.2 in children with two symptoms to 8.8 with three and 11.5 with four. Correlations are summarized in Table 4.

### 3.5. Bland–Altman Agreement Analysis

Because rank correlation quantifies ordinal ranking but not interchangeability, a Bland–Altman analysis was performed after normalizing the endoscopy and CT scores to a percentage of their respective maxima. The mean difference (endoscopy minus CT) was −10.6 percentage points, indicating that endoscopy systematically underestimates disease severity relative to CT (one-sample t = −8.0, *p* < 0.0001). The 95% limits of agreement were wide, from −32.6 to +11.4 percentage points, with 68 of 73 patients (93.2%) falling within these limits (Figure 6). A mild negative correlation between the mean of the two scores and their difference (r = −0.34) indicated a proportional bias: endoscopy underestimated CT severity more strongly at higher disease burdens. Together, these findings show that although endoscopy ranks children by severity closely, the two scales cannot be regarded as interchangeable at the individual level.

### 3.6. Disease Subgroups: Polyps and Septal Deviation

Children with at least one polyp-positive side (*n* = 7) had higher CT severity than those without polyps (median LMS 9 vs. 6, Mann–Whitney *p* = 0.007) and higher endoscopy totals (mean 4.6 vs. 2.2). The side-specific endoscopy–CT correlation was robust in both subgroups (polyp: ρ = 0.70; non-polyp: ρ = 0.61). Significant nasal septal deviation was identified in 34 children (46.6%); these children had marginally higher CT severity than those without septal deviation (median LMS 8 vs. 6, *p* = 0.045), with no significant difference in endoscopy score (*p* = 0.31). Although septal deviation may modestly aggravate sinus disease, it was not a confounder of the endoscopy–CT relationship.

### 3.7. Adenoid Size and Comorbidities

Adenoid size was unrelated to CT severity (vs LMS ρ = −0.00, *p* = 0.99; vs. MLMS ρ = 0.02) and, with graded endoscopic scoring, showed no significant correlation with the endoscopy score (ρ = 0.18, *p* = 0.13). As expected, adenoid size decreased with age (ρ = −0.40, *p* < 0.001) and correlated weakly and inversely with symptom count (ρ = −0.25, *p* = 0.031). Asthma and allergy were not significantly associated with CT severity (Mann–Whitney *p* = 0.10 and *p* = 0.10, respectively). Children with documented passive smoke exposure unexpectedly had lower CT severity (median LMS 5 vs. 8, *p* = 0.003) and lower endoscopy scores (mean 1.5 vs. 2.9, *p* = 0.001) than unexposed children; this finding is descriptive only, was not pre-specified and may reflect uncontrolled confounding (for example, by age or referral pattern) rather than a protective effect of exposure.

### 3.8. Histopathology and Disease Severity

Mucosal histopathology was eosinophilic in 9 children, neutrophilic in 20 and nonspecific in 43 (one specimen showed a predominantly plasmacytic infiltrate). CT severity differed significantly across these groups (Kruskal–Wallis *p* = 0.008): the median LMS was nine in eosinophilic, seven in neutrophilic and five in nonspecific disease. Endoscopy scores followed the same gradient (*p* = 0.022), and nasal polyps were almost confined to eosinophilic disease (67% of eosinophilic cases vs. 5% neutrophilic and 0% nonspecific) (Table 5). The eosinophilic subgroup was small (*n* = 9), so these associations should be interpreted cautiously.

### 3.9. Anatomical Variants

The most common lateral nasal wall variant was the agger nasi cell (30 children, 41.1%), followed by septal deviation (34, 46.6%), concha bullosa (20, 27.4%), the Onodi cell (13, 17.8%) and the Haller cell (12, 16.4%). Across the eight variant–sinus comparisons, only the agger nasi cell was associated with ipsilateral disease: on the right side, it correlated with frontal sinus severity (ρ = 0.39, raw *p* = 0.002), an association that remained significant after Benjamini–Hochberg correction (adjusted *p* = 0.017). The corresponding left-sided association did not reach significance (ρ = 0.25, adjusted *p* = 0.24). Concha bullosa, Haller cells and Onodi cells showed no association with inflammation of the related sinus (Table 6).

### 3.10. The Modified Lund–Mackay Score

Eighteen children had at least one underdeveloped sinus and therefore an MLMS exceeding their LMS; the two scales were almost perfectly correlated (ρ = 0.99) and reclassified no patient across the LMS ≥ 5 threshold. The MLMS correlated with the endoscopy score no more closely than the LMS (ρ = 0.76 vs. 0.76; Williams’ test *p* = 0.81). The study was not specifically powered to detect small differences between the two scales, and the MLMS may still be relevant in younger cohorts with less complete sinus pneumatization.

## 4. Discussion

In this cohort of children with surgically managed CRS, a graded nasal endoscopic score correlated strongly with CT severity. The association held both at the patient level (ρ = 0.76) and side-specifically (ρ = 0.63), and all three endoscopic signs—mucosal edema most strongly—correlated with ipsilateral CT disease severity. However, a Bland–Altman analysis showed a systematic underestimation by endoscopy relative to CT of about 10 percentage points, with wide 95% limits of agreement (−32.6% to +11.4%) and a mild proportional bias. Strong ordinal correlation and modest quantitative agreement therefore coexist: endoscopy ranks disease severity closely but does not measure the same numerical quantity as CT.

### 4.1. Comparison with Prior Literature

A correlation between structured endoscopic scores and CT severity has been reported in adults, generally with moderate-to-strong Spearman coefficients. Krishniya et al. [19], for example, reported a strong correlation between the Lund–Kennedy and Lund–Mackay scores in adult chronic rhinosinusitis undergoing functional endoscopic sinus surgery. Direct pediatric evidence remains limited. Bhattacharyya et al. examined the diagnostic accuracy of CT in pediatric CRS [10] and Hill et al. quantified the high prevalence of incidental abnormalities on pediatric sinus imaging [8], but neither paired graded endoscopy with CT scoring side by side. Consensus guidance in children [20] emphasizes the combination of symptoms, endoscopic examination and imaging but does not quantify their concordance. Our patient-level correlation (ρ = 0.76) is at the upper end of the adult range reported by Krishniya et al. [19], but the wide Bland–Altman limits of agreement suggest that the numerical closeness of the two scores has been overstated by correlation coefficients alone. Reporting both correlation and Bland–Altman analysis is therefore important, and the side-specific analysis in this study adds a further clinical dimension: endoscopy captures the lateralization of disease, not merely its overall burden.

### 4.2. Can Endoscopy Replace CT in Treatment Decisions?

The combined findings provide a nuanced answer. For grading severity in a given child at a given time, a structured Lund–Kennedy score reflects CT severity well as an ordinal measure and, because it involves no ionizing radiation, is well suited to repeated assessment during follow-up and monitoring the response to medical therapy. For the initial diagnosis of CRS, an endoscopic examination together with compatible symptoms already satisfies the EPOS criteria without a CT scan [3]. In these settings, endoscopy may reasonably be used in place of imaging, thereby reducing radiation exposure in a radiosensitive population.

For decisions about surgical management, however, endoscopy cannot substitute for CT. Endoscopy cannot visualize the sphenoid or posterior ethmoid sinuses, does not delineate the ostiomeatal complex in three dimensions and cannot identify dangerous anatomical variants such as an Onodi cell in relation to the optic canal [21,22]. Furthermore, the Bland–Altman analysis shows that endoscopy underestimates CT severity systematically and with wide limits of agreement, so an endoscopy score in isolation cannot be relied upon to quantify the true anatomical extent of disease. CT therefore remains necessary before functional endoscopic sinus surgery in children, both for anatomical mapping and for safety. The most consistent interpretation of our data is that endoscopy and CT are complementary rather than interchangeable: endoscopy for triage, grading and follow-up and CT when surgical planning or complex anatomy is at issue.

### 4.3. Adenoid, Comorbidities and Score Discrimination

Symptom burden correlated only moderately with CT severity—more weakly than endoscopy did. Symptoms in children are non-specific and overlap with adenoid disease, allergy and viral infection [3,4]; they are essential for deciding whom to investigate but are a poor proxy for the extent of sinus inflammation. Adenoid size, in turn, was unrelated to CT severity and, once endoscopy was graded rather than scored as merely present or absent, showed no significant association with the endoscopic findings. The adenoid may still contribute to pediatric CRS as a reservoir of infection [4,7], but its size alone does not indicate the severity of sinus inflammation. Of the recorded comorbidities, asthma and allergy were not significantly associated with CT severity; the apparent association of lower CT severity with documented passive smoke exposure is counterintuitive, was not pre-specified and is most plausibly attributable to uncontrolled confounding such as referral pattern or age.

Children with polyps had higher CT severity and higher endoscopy scores than those without, and the side-specific endoscopy–CT correlation was preserved in both subgroups, indicating that the concordance is not an artifact of polyp-positive cases alone. Significant septal deviation, present in nearly half of the cohort, was associated with marginally higher CT severity but did not alter the endoscopy–CT relationship.

### 4.4. Observer Bias and Selection

Observer bias is the most important methodological limitation. A single examiner performed both the CT review and the nasal endoscopy without formal blinding; the CT scans were clinically reviewed before the endoscopy was undertaken, so knowledge of the imaging findings could have influenced endoscopic scoring (and, in principle, vice versa). Expectation bias of this kind could inflate the apparent concordance, and the magnitude of the endoscopy–CT association should therefore be confirmed in prospective studies that score the two modalities independently and blind to each other. Topical anesthesia is unlikely to have contributed: lignocaine was applied as a gel on the endoscope itself rather than as a mucosal spray, limiting direct contact with the nasal mucosa. A second important limitation is selection: this cohort comprised children with disease severe enough to require surgical referral, so the endoscopic prevalence was high (94.5%) and the predictive performance of endoscopy in milder, medically managed populations may differ considerably.

### 4.5. Histopathology, Variants and the MLMS

The histopathological findings add a further layer. Eosinophilic and, to a lesser extent, neutrophilic mucosal inflammation were associated with greater CT severity and higher endoscopy scores than nonspecific inflammation, and polyps were almost confined to eosinophilic disease. Because the eosinophilic subgroup contained only nine patients, these associations should be interpreted cautiously, but mucosal endotype appears to contribute information beyond that provided by imaging or endoscopy alone.

The analysis of anatomical variants refines, rather than overturns, the existing literature. The prevalence of variants in this cohort was consistent with previous pediatric series [23,24,25,26,27,28]. Of the five variants examined, only the agger nasi cell was associated with disease in the related sinus—specifically with right frontal sinusitis—and this association survived correction for multiple comparisons. The agger nasi cell lies in the frontal drainage pathway, so a mechanical contribution to frontal outflow obstruction is biologically plausible. The absence of any association for concha bullosa, Haller cells and Onodi cells agrees with several pediatric studies [24,27] and supports the view that, in the growing facial skeleton, these variants are not yet large enough to obstruct sinus drainage; earlier reports of a positive association, particularly for concha bullosa, derive mainly from adult populations [29,30]. The modified Lund–Mackay score performed comparably to the conventional scale; it did not correlate more closely with endoscopic disease and reclassified no patient across the diagnostic threshold but may still be useful in younger cohorts with a higher proportion of underdeveloped sinuses.

### 4.6. Strengths and Limitations

The strengths of this study include the simultaneous collection of CT, endoscopic, symptom, adenoid and histopathological data in a well-defined cohort, the use of a recognized graded endoscopic system scored separately for each side, the use of cluster bootstrapping, multiple-comparison correction and Bland–Altman analysis, and pre-specified attention to score discrimination. Several limitations should be acknowledged. The study was retrospective and single-center, and the cohort comprised children whose disease was severe enough to require surgery, so the findings—including the strength of the endoscopy–CT correlation—may not generalize to milder, medically managed CRS. CT and endoscopy were performed and scored by one unblinded examiner, which may have inflated the observed concordance and precludes any estimate of inter-observer reliability. The interval between CT and endoscopy varied across patients and dates were not retrievable, so the effect of interval on concordance could not be examined. Symptom data were extracted retrospectively from records and were a simple count of four symptoms rather than a validated patient-reported outcome measure. The per-side CT sub-score treated an absent sinus as contributing zero, which slightly lowers the score of sides with sinus aplasia. The study lacked a healthy comparison group and was not specifically powered to detect small differences between the LMS and MLMS. Prospective, multicenter studies with blinded, independent scoring of the two modalities, a validated pediatric symptom instrument and inclusion of milder disease are needed to confirm the strength of the endoscopy–CT association and its utility for triaging imaging.

## 5. Conclusions

In children with surgically managed chronic rhinosinusitis, a graded nasal endoscopic score correlated strongly with CT severity, both overall and side-specifically, but Bland–Altman analysis showed a systematic underestimation and wide limits of agreement, so the two scales are not quantitatively interchangeable at the individual level. A structured endoscopic score is therefore a useful, non-ionizing surrogate measure of the severity and lateralization of sinus disease and can help decide which children require CT, although it is best interpreted as a graded score and does not replace CT for surgical planning. Because the cohort comprised children with advanced disease, the strong concordance observed here may not extend to milder CRS. Among anatomical variants, only the agger nasi cell was associated with ipsilateral (frontal) sinus disease, and the modified Lund–Mackay score offered no measurable advantage over the conventional scale in this cohort.

## Figures and Tables

**Table 1:** Frequency of inflammatory involvement of the paranasal sinuses (*n* = 73).

Sinus	Right Only	Left Only	Bilateral	Total, *n* (%)
**Maxillary**	13	12	43	68 (93.2)
**Anterior ethmoid**	14	11	38	63 (86.3)
**Posterior ethmoid**	14	11	16	41 (56.2)
**Sphenoid**	7	11	13	31 (42.5)
**Frontal**	11	4	10	25 (34.2)

Involvement defined as a Lund–Mackay sub-score > 0 on the side concerned.

**Table 2:** Endoscopic, adenoid, symptom and comorbidity findings (*n* = 73).

Assessment	Finding	Value
**Endoscopy**	Mucosal edema, *n* (%)	65 (89.0)
	Mucopurulent discharge, *n* (%)	40 (54.8)
	Nasal polyps, *n* (%)	7 (9.6)
	Lund–Kennedy total score, mean ± SD	2.5 ± 1.7
	Endoscopy-positive (score ≥ 1), *n* (%)	69 (94.5)
**Adenoid**	Choanal obstruction, mean ± SD	26.3 ± 19.8%
	Obstruction ≥ 50%, *n* (%)	12 (16.4)
**Symptoms**	Nasal discharge, *n* (%)	64 (87.7)
	Nasal obstruction, *n* (%)	47 (64.4)
	Headache/facial pressure, *n* (%)	44 (60.3)
	Cough, *n* (%)	28 (38.4)
**Comorbidities**	Asthma, *n* (%)	6 (8.2)
	Allergy, *n* (%)	13 (17.8)
	Passive smoke exposure, *n* (%)	22 (30.1)

**Table 3:** Side-specific agreement between nasal endoscopy and CT at increasing CT thresholds (146 nasal sides).

CT Threshold for a Diseased Side	CT-Positive Sides, *n*	Agreement, %	Cohen’s κ	Sensitivity, %	Specificity, %
**Sub-score ≥ 1**	124	71.9	0.21	75	55
**Sub-score ≥ 2**	110	74.7	0.36	80	58
**Sub-score ≥ 3**	90	77.4	0.50	89	59

An endoscopy-positive side was defined as a Lund–Kennedy side score ≥1. The CT sub-score is the sum of the five sinus Lund–Mackay sub-scores on that side (range 0–10). Sensitivity and specificity are for endoscopy against CT.

**Table 4:** Spearman correlations among nasal endoscopy, CT severity and symptom burden.

Comparison	ρ (Rho)	95% CI	*p*-Value
**Per-side endoscopy vs. CT sub-score (146 sides)**	0.63	0.50 to 0.74	<0.0001
**Endoscopy total vs. LMS (patient)**	0.76	0.63 to 0.86	<0.0001
**Endoscopy total vs. MLMS (patient)**	0.76	0.64 to 0.85	<0.0001
**Symptom count vs. LMS**	0.41	0.20 to 0.59	0.0003
**Symptom count vs. endoscopy total**	0.43	0.21 to 0.62	0.0002

CI, confidence interval; LMS, Lund–Mackay scale; MLMS, modified Lund–Mackay scale. The per-side interval was obtained by cluster bootstrap resampling patients and patient-level intervals by ordinary bootstrap.

**Table 5:** Disease severity by predominant mucosal inflammatory pattern.

Histopathology	*n*	Median LMS	Endoscopy Score, Mean	Polyps, %
**Eosinophilic**	9	9	3.6	67
**Neutrophilic**	20	7	2.8	5
**Nonspecific**	43	5	2.1	0

Kruskal–Wallis test: LMS *p* = 0.008; endoscopy score *p* = 0.022.

**Table 6:** Correlation of anatomical variants with severity of inflammation in the related sinus.

Variant and Sinus	ρ (Rho)	Raw *p*	Adjusted *p*
**Agger nasi vs. frontal (right)**	0.39	0.002	0.017
**Agger nasi vs. frontal (left)**	0.25	0.059	0.24
**Concha bullosa vs. maxillary (right)**	0.03	0.81	0.81
**Concha bullosa vs. maxillary (left)**	−0.04	0.72	0.81
**Haller cell vs. maxillary (right)**	0.04	0.76	0.81
**Haller cell vs. maxillary (left)**	−0.07	0.56	0.81
**Onodi cell vs. sphenoid (right)**	0.12	0.34	0.81
**Onodi cell vs. sphenoid (left)**	0.05	0.68	0.81

Adjusted *p*-values: Benjamini–Hochberg correction across the eight comparisons. Sides with an absent sinus were excluded from the relevant correlations.

## Data Availability

The data supporting the findings of this study are available from the corresponding author upon reasonable request.

## Abbreviations

The following abbreviations are used in this manuscript:

ARS	acute rhinosinusitis
CRS	chronic rhinosinusitis
CT	computed tomography
EPOS	European Position Paper on Rhinosinusitis and Nasal Polyps
LMS	Lund–Mackay scale
MLMS	modified Lund–Mackay scale
